# Wt p53 impairs response to chemotherapy: make lemonade to spare normal cells

**DOI:** 10.18632/oncotarget.548

**Published:** 2012-07-13

**Authors:** Mikhail V. Blagosklonny

**Affiliations:** ^1^ Department of Cell Stress Biology, Roswell Park Cancer Institute, Buffalo, NY, USA

**Keywords:** cancer, DNA damaging drugs, side effects, apoptosis, senescence, drug combinations, cyclotherapy

## Abstract

As published recently in *Cancer Cell*, p53 impairs the apoptotic response to chemotherapy and clinical outcome in breast cancer. I discuss that, while treating tumors lacking wt p53, this phenomenon can be exploited to protect normal cells from chemotherapy because all normal cells have wt p53. Also, several therapeutic paradigms can be reassessed, including the role of cellular senescence in cancer therapy.

## Background information

By demonstrating that p53-mediated senescence impairs the apoptotic response to doxorubicin (Dox), a DNA damaging and p53-inducing drug, Lozano and co-authors suggest the reassessment of the paradigm for p53 in cancer therapy [1]. Taking up this invitation, I will discuss how to spare normal cells from chemotherapy, while eliminating cancer cells lacking wt p53.

In cell culture, any cancer cell can be killed by chemotherapy. But chemotherapy poorly discriminates cancer and normal cells. It damages both types of cells. Side effects limit the therapy, precluding the elimination of cancer cells(see appendix footnote 1). If we could protect vital normal tissues from chemotherapy without protecting cancer cells, then we could cure cancer.

Although this topic is not addressed by Lozano and co-workers, the paper hints on the solution. As described, doxorubicin-treated p53 mutant tumors failed to arrest proliferation, leading to abnormal mitoses and cell death, whereas p53 wild-type tumors arrested, avoiding mitotic catastrophe and cell death [1]. In tumors lacking p53, this can be exploited for selective protection of normal cells from chemotherapy. Let us see how.

## From drug to drug combination

**Figure 1:**
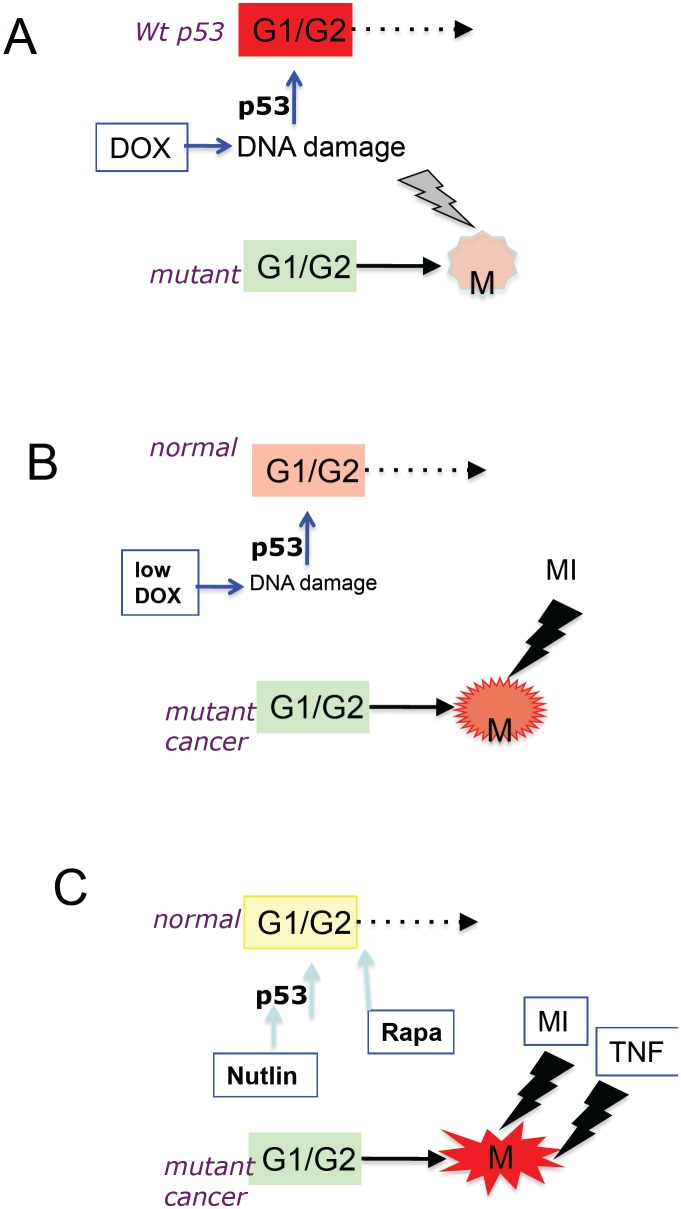
Protection of normal cells: From a single drug to ordered combinations (A) Doxorubicin (Dox) causes G1/G2 arrest (red) in wt p53 cells, whereas cancer cells with mutant p53 enter mitosis (M) and undergo mitotic catastrophe. (B) Low doses of doxorubicin (low DOX) cause a more gentle G1/G2 arrest (orange) in normal cells, whereas cancer cells with mutant p53 enter mitosis (M) and are killed by MI (mitotic inhibitor such as Taxol). (C) Nutlin-3a plus rapamycin cause the gentlest G1/G2 arrest (yellow) in normal cells, whereas cancer cells with mutant p53 enter mitosis (M) and are killed by a highly apoptotic combination of MI plus TRAIL or TNF.

Since all normal cells have wt p53, its induction by Doxorubicin can in theory protect normal cells from the cytotoxicity of Doxorubicin itself. In contrast, tumor cells with mutant p53 will be selectively killed (Fig. 1 A). Yet, the selectivity is difficult to achieve by using one drug. To do so, Doxorubicin should arrest normal cells without permanently damaging them. Therefore, doses of Doxorubicin should be low, just sufficient to induce p53 in normal cells. But at such doses, the damage may be not sufficient to cause mitotic catastrophe in most (or any) cancer cells. If the dose is increased, there will be harm to normal cells. Doxorubicin cannot protect and kill efficiently at the same dose.

The solution is to use two drugs (Fig. 1B): the first drug will induce p53 and the second drug will cause catastrophe during mitosis [2, 3]. Then, as Russian proverb goes, both “the wolves are sated, and the sheep are intact.” Low doses of Doxorubicin, which arrest cells with wt p53, do not arrest cells lacking wt p53. Cells lacking wt p53 enter mitosis. If such cells are treated with mitotic inhibitors (MI), then they, for sure, undergo mitotic catastrophe. In apoptosis-prone cells, this culminates in apoptosis (see appendix, footnote 2).

As a p53-inducing drug, Doxorubicin can be substituted with low doses of etoposide, actinomycin D and leptomycin. In cell culture, these agents protected normal cells as well as cancer cells with wt p53 from mitotic inhibitors (MI) such as paclitaxel, docetaxel, vinblastine and nocodazole as well as inhibitors of mitotic kinases [2-10]. Especially Actinomycin D could be used at extremely low concentrations [6, 8].Still, even at low doses, DNA-damaging p53-inducing drugs may be viewed as too damaging. Also, they may arrest certain types of cancer cells by p53-independent mechanism, requiring the inclusion of Chk1 inhibitors in the combination [11]. DNA damaging drugs can be substituted by Mdm2 inhibitors such as nutlin-3a and MI-219, which induce p53, without DNA damage [12-15]. Mdm2 inhibitors can be used for protection of normal cells during chemotherapy of tumors lacking p53. Nutlin-3a selectively protects cells with wt p53 [7, 10, 16-18]. In animals, Mdm2 inhibitors do not have dose-limiting side effects [14, 19, 20]. And most importantly, nutlin-3a decreases side effects of chemotherapy in mice [20]. The main disadvantage of Mdm2-inhibtors is that they are not approved yet for clinical use. Nutlin-3a analogs are undergoing clinical trials as anti-cancer drugs not as protective agents. They may (or may not) fail as anticancer drugs. But their utility, as protective drugs, is valuable. P53 may cause senescence and reversible quiescence [21-28]. A brief treatment with Nutlin-3a induces reversible arrest in most normal cells [29, 30] (see appendix, footnote 3). Still, to avoid senescence caused by nutlin-3a in some cell types, rapamyin can be added to the drug combination (Fig. 1C). Not only does rapamycin suppress the conversion from reversible arrest to senescence [22, 31] but it also can protect normal cells from chemotherapy [17, 32]. When normal cells are firmly protected, then additional drugs can be added to the combination to increase apoptosis in cancer cells (Fig. 1C). For example, cancer cells arrested in mitosis by MI become extremely sensitive to TNF and TRAIL. During mitosis, transcription is inhibited and this sensitizes cells to TNF and TRAIL. When transcription is inhibited, TNF can induce apoptosis even in the most apoptosis-reluctant cells [33], a goal of cancer therapy (see the next section). An example of cyclotherapeutic combination (footnote 4) containing 4 drugs is: nutlin-3a + rapamycin followed by paclitaxel + TRAIL.

Furthermore, the strategy to protect normal cells is not limited to targeting wt p53 (or loss of p53). It may involve other targets present or absent in cancer cells [34, 35]. For example, Rb is often lost in human cancer, rendering cells resistant to arrest caused by CDK4/6 inhibitors [36]. Yet, CDK4/6 inhibitors arrest normal cells. Therefore, co-administration of PD0332991 (a CDK4/6 inhibitor) plus carboplatin provided protection of bone marrow, without protection of Rb-deficient tumors [37]. Similarly, administration of PD0332991 to mice reduced treatment toxicity of radiation without compromising the therapeutic tumor response [38, 39]. Loss of Rb and p53 coincided in the most aggressive cancers [40, 41], rendering them a perfect target for cyclotherapy. Also, fasting can selectively protect normal cells from chemotherapy and decrease side effects in patients [42-45]. Among other effects, fasting inhibits the nutrient-sensing mTOR pathway [46]. Noteworthy, a combination of rapamycin and metformin protected normal cells from MI [17]. Furthermore, the combination of a protective drug (a substrate of Pgp/MRP) plus a cytotoxic drug (not a substrate) selectively kills multidrug resistant cancer cells, while sparing non-resistant cells [4, 47-49]. Finally, there are several other strategies of selective protection of normal cells [50-58].

## Combining letters in words, words in phrases

It is difficult enough to treat cancer. Why should this be done in the hardest way: by using a single drug and scrambled combinations, instead of cyclotherapeutic and other ordered combinations? In analogy, it is difficult to write poems like Pushkin, for instance, but absolutely impossible to write them by using one letter. By using at least 2 “letters”, we can increase a therapeutic window. And by using 4 or 5, one can design effective and selective therapy. Still, p53-dependent cyclotherapy will eliminate cells with mutant p53 while sparing cancer cells with wt p53, thus selecting for wt p53 tumors. And such therapy cannot be used against wt p53 cancers to start with. Of course ordered combinations are not limited to targeting p53 [34, 35, 48].

One general solution is alternating super-combinations: ordered (cyclotherapeutic) combinations can be alternated with other modalities such as conventional therapy, cancer- and tissue- selective drugs [59-63]. For example, nutlin-3 is cytotoxic to cancer cells with wt p53 [19, 64, 65]. While causing response, nutlin-3a (as any drug) will select for drug (nutlin) resistance. In fact, nutlin-3a selects for mutant p53 [66]. Then the therapy with nutlin-3a can be followed by cyclotherapeutic combination. This alternating strategy was recently discussed in detail. Like letters (drugs) can be combined in words (ordered combinations), the words can be combined in phrases.

## Cell senescence is not first choice

An important point to reassess is whether senescence is a goal of cancer therapy. Fifteen years ago, it was accepted that it is apoptosis that is a goal [67-70]. In fact, chemotherapy induces apoptosis in curable malignancies such as leukemia, lymphoma and childhood cancers. Unfortunately, most common cancers are apoptosis-reluctant. In most common cancers, apoptosis is not a predictive marker of the therapeutic response. However, one may argue that these cancers are poorly curable exactly because chemotherapy does not induce apoptosis. So it is not that apoptosis is not a goal, simply it is not easily achievable in the majority of cancers. (Note: Still certain drug combinations cause apoptosis in the most apoptosis-reluctant cells. These combinations could be used when normal cells are protected as shown in figure 1C). In apoptosis-reluctant cells, standard chemotherapy cause either slow cell death or senescence (at low drug concentrations). Therefore, it was suggested that senescence is a feasible goal [71, 72], perhaps because it can by induced in common cancers by almost any cytostatic drug. Conventional anti-cancer drugs at low concentrations all induce senescence in cell culture but do not cure cancer patients. Either they do not induce senescence in the organism or senescence is not sufficient or both.

The paper by Jackson *et al* demonstrated that senescence is not effective, whereas apoptosis is effective [1]. Furthermore, a senescent phenotype including hyper-secretory and pro-inflammatory features seems to be counter-productive [1], in agreement with an existing paradigm [71, 73]. Senescence is a form of cell-cycle arrest, when oncogenic and growth-promoting pathways are still active [74]. Whereas cell-cycle arrest is a barrier to cancer growth (by definition), a senescent phenotype (due to over-activation of growth-promoting pathways in arrested cells) is not [74]. Two therapeutic approaches can be suggested. First, senescent cells can be further targeted [75]. Second, rapamycin can partially suppress the senescent phenotype without abrogating cell cycle arrest [31]. If the senescent phenotype is not of any importance for anti-cancer therapy, co-treatment with rapamycin will not prevent antitumor effect of doxorubicin. This could be tested in the Lozano model [1]. Noteworthy, rapamycin can be employed to prevent senescence of normal cells (Fig. 1C) and cancer cells. And the same modalities (rapamycin, metformin, nutlin-3a, fasting) can be used for protection of normal cells and for cancer prevention [76-80]. And calorie restriction and rapamycin extend lifespan in diverse species [81-83]. This may be not a co-incidence. But this is a topic for another article.

## Appendix: Footnotes

### Footnote 1

In cancer patients only a few types of cancer are curable by chemotherapy alone. Curable cancers arise from tissues prone to apoptosis such as lymphoid, testicular, embryonic and placental/endometrial. For example, testicular germ cell tumors with wt p53 are very sensitive to p53-inducing chemotherapy [84, 85]. Following therapy, relapsed tumors often lack wt p53 and are resistant to therapy [86-88]. Most common (aging-related) cancers such as breast, prostate, colon, gastric, thyroid, pancreatic, lung are hardly curable by chemotherapy, because they are not more sensitive to chemotherapy than normal cells are. In such cases, chemotherapy cannot eliminate cancer cells, without destroying normal cells of vital tissues.

### Footnote 2

Cells can be apoptosis-prone and apoptosis-reluctant [89], which in part determines cell fate following mitotic arrest [90]. These effects varied dramatically depending on the drug and cell line [91]. In general, p53 is not a marker of resistance to therapy because apoptosis-prone tumors tend to lose p53 (to avoid apoptosis), whereas apoptosis-reluctant cancers may retain wt p53 [92].

### Footnote 3

The choice between quiescence and senescence is determined in part by the activity of the nutrient-sensing, growth-promoting mTOR pathway [24-28, 93-96]. When the cell cycle is arrested (by any means) but growth-promoting pathways are not, then cells become senescent. Rapamycin decelerates geroconversion (the conversion from reversible arrest to senescence) [22, 31]. By inhibiting gerogenic pathways such as mTOR, nutlin-3a by itself can suppress senescence, thus causing reversible arrest instead [21-23].

### Footnote 4

Cyclotherapeutic and other ordered combinations are antagonistic in normal cells [97]. So far, cyclotherapeutic combinations were not tested in the clinic, albeit drugs that could be used in cyclotherapetic combinations are used in conventional (scrambled) combinations. In conventional (scrambled) combinations, each drug is intended to damage cells, not to selectively protect normal cells. Drugs are combined for synergy usually at full doses, thus increasing side effects. In contrast in cyclotherapeutic (ordered) combinations, the choice of drugs, doses and sequences are the key.

## References

[1] Jackson JG, Pant V, Li Q, Chang LL, Quintas-Cardama A, Garza D, Tavana O, Yang P, Manshouri T, Li Y, El-Naggar AK, Lozano G (2012). p53-Mediated Senescence Impairs the Apoptotic Response to Chemotherapy and Clinical Outcome in Breast Cancer. Cancer Cell.

[2] Blagosklonny MV, Robey R, Bates S, Fojo T (2000). Pretreatment with DNA-damaging agents permits selective killing of checkpoint-deficient cells by microtubule-active drugs. J Clin Invest.

[3] Blagosklonny MV, Pardee AB (2001). Exploiting cancer cell cycling for selective protection of normal cells. Cancer Res.

[4] Demidenko ZN, Halicka D, Kunicki J, McCubrey JA, Darzynkiewicz Z, Blagosklonny MV (2005). Selective killing of adriamycin-resistant (G2 checkpoint-deficient and MRP1-expressing) cancer cells by docetaxel. Cancer Res.

[5] Demidenko ZN, Vivo C, Halicka HD, Li CJ, Bhalla K, Broude EV, Blagosklonny MV (2006). Pharmacological induction of Hsp70 protects apoptosis-prone cells from doxorubicin: comparison with caspase-inhibitor- and cycle-arrest-mediated cytoprotection. Cell Death Differ.

[6] Choong ML, Yang H, Lee MA, Lane DP (2009). Specific activation of the p53 pathway by low dose actinomycin D: a new route to p53 based cyclotherapy. Cell Cycle.

[7] Cheok CF, Kua N, Kaldis P, Lane DP (2010). Combination of nutlin-3 and VX-680 selectively targets p53 mutant cells with reversible effects on cells expressing wild-type p53. Cell Death Differ.

[8] Rao B, van Leeuwen IM, Higgins M, Campbel J, Thompson AM, Lane DP, Lain S (2010). Evaluation of an Actinomycin D/VX-680 aurora kinase inhibitor combination in p53-based cyclotherapy. Oncotarget.

[9] van Leeuwen IM, Lain S (2011). Pharmacological manipulation of the cell cycle and metabolism to protect normal tissues against conventional anticancer drugs. Oncotarget.

[10] van Leeuwen IM, Rao B, Sachweh MC, Lain S (2012). An evaluation of small-molecule p53 activators as chemoprotectants ameliorating adverse effects of anticancer drugs in normal cells. Cell Cycle.

[11] Blagosklonny MV (2002). Sequential activation and inactivation of G2 checkpoints for selective killing of p53-deficient cells by microtubule-active drugs. Oncogene.

[12] Vassilev LT (2004). Small-molecule antagonists of p53-MDM2 binding: research tools and potential therapeutics. Cell Cycle.

[13] Vassilev LT (2005). p53 Activation by small molecules: application in oncology. J Med Chem.

[14] Shangary S, Qin D, McEachern D, Liu M, Miller RS, Qiu S, Nikolovska-Coleska Z, Ding K, Wang G, Chen J, Bernard D, Zhang J, Lu Y, Gu Q, Shah RB, Pienta KJ (2008). Temporal activation of p53 by a specific MDM2 inhibitor is selectively toxic to tumors and leads to complete tumor growth inhibition. Proc Natl Acad Sci U S A.

[15] Popowicz GM, Czarna A, Wolf S, Wang K, Wang W, Domling A, Holak TA (2010). Structures of low molecular weight inhibitors bound to MDMX and MDM2 reveal new approaches for p53-MDMX/MDM2 antagonist drug discovery. Cell Cycle.

[16] Carvajal D, Tovar C, Yang H, Vu BT, Heimbrook DC, Vassilev LT (2005). Activation of p53 by MDM2 antagonists can protect proliferating cells from mitotic inhibitors. Cancer Res.

[17] Apontes P, Leontieva OV, Demidenko ZN, Li F, Blagosklonny MV (2011). Exploring long-term protection of normal human fibroblasts and epithelial cells from chemotherapy in cell culture. Oncotarget.

[18] van Leeuwen IM, Higgins M, Campbell J, Brown CJ, McCarthy AR, Pirrie L, Westwood NJ, Lain S (2011). Mechanism-specific signatures for small-molecule p53 activators. Cell Cycle.

[19] Tovar C, Rosinski J, Filipovic Z, Higgins B, Kolinsky K, Hilton H, Zhao X, Vu BT, Qing W, Packman K, Myklebost O, Heimbrook DC, Vassilev LT (2006). Small-molecule MDM2 antagonists reveal aberrant p53 signaling in cancer: implications for therapy. Proc Natl Acad Sci U S A.

[20] Sur S, Pagliarini R, Bunz F, Rago C, Diaz LA, Kinzler KW, Vogelstein B, Papadopoulos N (2009). A panel of isogenic human cancer cells suggests a therapeutic approach for cancers with inactivated p53. Proc Natl Acad Sci U S A.

[21] Demidenko ZN, Korotchkina LG, Gudkov AV, Blagosklonny MV (2010). Paradoxical suppression of cellular senescence by p53. Proc Natl Acad Sci U S A.

[22] Korotchkina LG, Leontieva OV, Bukreeva EI, Demidenko ZN, Gudkov AV, Blagosklonny MV (2010). The choice between p53-induced senescence and quiescence is determined in part by the mTOR pathway. Aging (Albany NY).

[23] Leontieva O, Gudkov A, Blagosklonny M (2010). Weak p53 permits senescence during cell cycle arrest. Cell Cycle.

[24] Long JS, Ryan KM (2010). p53 and senescence: a little goes a long way. Cell Cycle.

[25] Santoro R, Blandino G (2010). p53: The pivot between cell cycle arrest and senescence. Cell Cycle.

[26] Serrano M (2010). Shifting senescence into quiescence by turning up p53. Cell Cycle.

[27] Darzynkiewicz Z (2010). Another “Janus paradox” of p53: induction of cell senescence versus quiescence. Aging (Albany NY).

[28] Lane DP, Verma C, Fang CC (2010). The p53 inducing drug dosage may determine quiescence or senescence. Aging (Albany NY).

[29] Huang B, Deo D, Xia M, Vassilev LT (2009). Pharmacologic p53 Activation Blocks Cell Cycle Progression but Fails to Induce Senescence in Epithelial Cancer Cells. Mol Cancer Res.

[30] Korotchkina LG, Demidenko ZN, Gudkov AV, Blagosklonny MV (2009). Cellular quiescence caused by the Mdm2 inhibitor nutlin-3a. Cell Cycle.

[31] Demidenko ZN, Zubova SG, Bukreeva EI, Pospelov VA, Pospelova TV, Blagosklonny MV (2009). Rapamycin decelerates cellular senescence. Cell Cycle.

[32] Darzynkiewicz Z (2011). Novel strategies of protecting non-cancer cells during chemotherapy: are they ready for clinical testing?. Oncotarget.

[33] Demidenko ZN, Blagosklonny MV (2004). Flavopiridol induces p53 via initial inhibition of Mdm2 and p21 and, independently of p53, sensitizes apoptosis-reluctant cells to tumor necrosis factor. Cancer Res.

[34] Blagosklonny MV (2003). Matching targets for selective cancer therapy. Drug Discov Today.

[35] Blagosklonny MV (2008). “Targeting the absence” and therapeutic engineering for cancer therapy. Cell Cycle.

[36] Dean JL, McClendon AK, Hickey TE, Butler LM, Tilley WD, Witkiewicz AK, Knudsen EK (2012). Therapeutic response to CDK4/6 inhibition in breast cancer defined by ex vivo analyses of human tumors. Cell Cycle.

[37] Roberts PJ, Bisi JE, Strum JC, Combest AJ, Darr DB, Usary JE, Zamboni WC, Wong KK, Perou CM, Sharpless NE (2012). Multiple roles of cyclin-dependent kinase 4/6 inhibitors in cancer therapy. J Natl Cancer Inst.

[38] Johnson SM, Torrice CD, Bell JF, Monahan KB, Jiang Q, Wang Y, Ramsey MR, Jin J, Wong KK, Su L, Zhou D, Sharpless NE (2010). Mitigation of hematologic radiation toxicity in mice through pharmacological quiescence induced by CDK4/6 inhibition. J Clin Invest.

[39] Gudkov AV, Komarova EA (2010). Radioprotection: smart games with death. J Clin Invest.

[40] Jiang Z, Jones R, Liu JC, Deng T, Robinson T, Chung PE, Wang S, Herschkowitz JI, Egan SE, Perou CM, Zacksenhaus E (2011). RB1 and p53 at the crossroad of EMT and triple-negative breast cancer. Cell Cycle.

[41] Ciavarra G, Zacksenhaus E (2011). Multiple pathways counteract cell death induced by RB1 loss: implications for cancer. Cell Cycle.

[42] Raffaghello L, Lee C, Safdie FM, Wei M, Madia F, Bianchi G, Longo VD (2008). Starvation-dependent differential stress resistance protects normal but not cancer cells against high-dose chemotherapy. Proc Natl Acad Sci U S A.

[43] Safdie FM, Dorff T, Quinn D, Fontana L, Wei M, Lee C, Cohen P, Longo VD (2009). Fasting and cancer treatment in humans: A case series report. Aging (Albany NY).

[44] Lee C, Raffaghello L, Brandhorst S, Safdie FM, Bianchi G, Martin-Montalvo A, Pistoia V, Wei M, Hwang S, Merlino A, Emionite L, de Cabo R, Longo VD (2012). Fasting cycles retard growth of tumors and sensitize a range of cancer cell types to chemotherapy. Sci Transl Med.

[45] Raffaghello L, Safdie F, Bianchi G, Dorff T, Fontana L, Longo VD (2010). Fasting and differential chemotherapy protection in patients. Cell Cycle.

[46] Blagosklonny MV (2010). Calorie restriction: Decelerating mTOR-driven aging from cells to organisms (including humans). Cell Cycle.

[47] Blagosklonny MV (1999). Drug-resistance enables selective killing of resistant leukemia cells: exploiting of drug resistance instead of reversal. Leukemia.

[48] Blagosklonny MV (2001). Treatment with inhibitors of caspases, that are substrates of drug transporters, selectively permits chemotherapy-induced apoptosis in multidrug-resistant cells but protects normal cells. Leukemia.

[49] Blagosklonny MV (2003). Targeting cancer cells by exploiting their resistance. Trends Mol Med.

[50] Komarov PG, Komarova EA, Kondratov RV, Christov-Tselkov K, Coon JS, Chernov MV, Gudkov AV (1999). A chemical inhibitor of p53 that protects mice from the side effects of cancer therapy. Science.

[51] Komarova EA, Gudkov AV (2001). Chemoprotection from p53-dependent apoptosis: potential clinical applications of the p53 inhibitors. Biochem Pharmacol.

[52] Strom E, Sathe S, Komarov PG, Chernova OB, Pavlovska I, Shyshynova I, Bosykh DA, Burdelya LG, Macklis RM, Skaliter R, Komarova EA, Gudkov AV (2006). Small-molecule inhibitor of p53 binding to mitochondria protects mice from gamma radiation. Nat Chem Biol.

[53] Burdelya LG, Krivokrysenko VI, Tallant TC, Strom E, Gleiberman AS, Gupta D, Kurnasov OV, Fort FL, Osterman AL, Didonato JA, Feinstein E, Gudkov AV (2008). An agonist of toll-like receptor 5 has radioprotective activity in mouse and primate models. Science.

[54] Burdelya LG, Gleiberman AS, Toshkov I, Aygun-Sunar S, Bapardekar M, Manderscheid-Kern P, Bellnier D, Krivokrysenko VI, Feinstein E, Gudkov AV (2011). Toll-like Receptor 5 Agonist Protects Mice from Dermatitis and Oral Mucositis Caused by Local Radiation: Implications for Head-and-Neck Cancer Radiotherapy. Int J Radiat Oncol Biol Phys.

[55] Du L, Smolewski P, Bedner E, Traganos F, Darzynkiewicz Z (2001). Selective protection of mitogenically stimulated human lymphocytes but not leukemic cells from cytosine arabinoside-induced apoptosis by LY294002, a phosphoinositol-3 kinase inhibitor. Int J Oncol.

[56] Kranz D, Dobbelstein M (2006). Nongenotoxic p53 activation protects cells against S-phase-specific chemotherapy. Cancer Res.

[57] Saha S, Bhanja P, Liu L, Alfieri AA, Yu D, Kandimalla ER, Agrawal S, Guha C (2012). TLR9 agonist protects mice from radiation-induced gastrointestinal syndrome. PLoS One.

[58] Blagosklonny MV, Bishop PC, Robey R, Fojo T, Bates SE (2000). Loss of cell cycle control allows selective microtubule-active drug-induced Bcl-2 phosphorylation and cytotoxicity in autonomous cancer cells. Cancer Res.

[59] Blagosklonny MV (2003). Tissue-selective therapy of cancer. Br J Cancer.

[60] Blagosklonny MV (2005). How Cancer Could Be Cured by 2015. Cell Cycle.

[61] Blagosklonny MV (2005). Overcoming limitations of natural anticancer drugs by combining with artificial agents. Trends Pharmacol Sci.

[62] Blagosklonny MV (2005). Teratogens as anti-cancer drugs. Cell Cycle.

[63] Blagosklonny MV (2011). NCI's provocative questions on cancer: some answers to ignite discussion. Oncotarget.

[64] Kojima K, Konopleva M, Samudio IJ, Shikami M, Cabreira-Hansen M, McQueen T, Ruvolo V, Tsao T, Zeng Z, Vassilev LT, Andreeff M (2005). MDM2 antagonists induce p53-dependent apoptosis in AML: implications for leukemia therapy. Blood.

[65] Cipriano R, Patton JT, Mayo LD, Jackson MW (2010). Inactivation of p53 signaling by p73 or PTEN ablation results in a transformed phenotype that remains susceptible to Nutlin-3 mediated apoptosis. Cell Cycle.

[66] Aziz MH, Shen H, Maki CG (2011). Acquisition of p53 mutations in response to the non-genotoxic p53 activator Nutlin-3. Oncogene.

[67] Fisher DE (1994). Apoptosis in cancer therapy: crossing the threshold. Cell.

[68] Martin SJ, Green DR (1994). Apoptosis as a goal of cancer therapy. Curr Opin Oncol.

[69] Lowe SW, Lin AW (2000). Apoptosis in cancer. Carcinogenesis.

[70] Blagosklonny MV (2000). Cell death beyond apoptosis. Leukemia.

[71] Roninson IB (2002). Tumor senescence as a determinant of drug response in vivo. Drug Resist Updat.

[72] Brown JM, Wilson G (2003). Apoptosis genes and resistance to cancer therapy: what does the experimental and clinical data tell us?. Cancer Biol Ther.

[73] Campisi J (2005). Senescent cells, tumor suppression, and organismal aging: good citizens, bad neighbors. Cell.

[74] Blagosklonny MV (2012). Cell cycle arrest is not yet senescence, which is not just cell cycle arrest: terminology for TOR-driven aging. Aging (Albany NY).

[75] Gudkov AV, Gurova KV, Komarova EA (2011). Inflammation and p53: A Tale of Two Stresses. Genes Cancer.

[76] Martin-Castillo B, Vazquez-Martin A, Oliveras-Ferraros C, Menendez JA (2010). Metformin and cancer: doses, mechanisms and the dandelion and hormetic phenomena. Cell Cycle.

[77] Anisimov VN (2010). Metformin for aging and cancer prevention. Aging (Albany NY).

[78] Anisimov VN, Egormin PA, Piskunova TS, Popovich IG, Tyndyk ML, Yurova MN, Zabezhinski MA, Anikin IV, Karkach AS, Romanyukha AA (2010). Metformin extends life span of HER-2/neu transgenic mice and in combination with melatonin inhibits growth of transplantable tumors in vivo. Cell Cycle.

[79] Blagosklonny MV (2008). Prevention of cancer by inhibiting aging. Cancer Biol Ther.

[80] Anisimov VN, Zabezhinski MA, Popovich IG, Piskunova TS, Semenchenko AV, Tyndyk ML, Yurova MN, Rosenfeld SV, Blagosklonny MV (2011). Rapamycin increases lifespan and inhibits spontaneous tumorigenesis in inbred female mice. Cell Cycle.

[81] Longo VD, Fontana L (2011). Intermittent supplementation with rapamycin as a dietary restriction mimetic. Aging (Albany NY).

[82] Blagosklonny MV (2010). Revisiting the antagonistic pleiotropy theory of aging: TOR-driven program and quasi-program. Cell Cycle.

[83] Blagosklonny MV (2011). Molecular damage in cancer: an argument for mTOR-driven aging. Aging (Albany NY).

[84] Chresta CM, Masters JR, Hickman JA (1996). Hypersensitivity of human testicular tumors to etoposide-induced apoptosis is associated with functional p53 and a high Bax:Bcl-2 ratio. Cancer Res.

[85] Li B, Cheng Q, Li Z, Chen J (2010). p53 inactivation by MDM2 and MDMX negative feedback loops in testicular germ cell tumors. Cell Cycle.

[86] Houldsworth J, Xiao H, Murty VV, Chen W, Ray B, Reuter VE, Bosl GJ, Chaganti RS (1998). Human male germ cell tumor resistance to cisplatin is linked to TP53 gene mutation. Oncogene.

[87] Gutekunst M, Oren M, Weilbacher A, Dengler MA, Markwardt C, Thomale J, Aulitzky WE, van der Kuip H (2011). p53 hypersensitivity is the predominant mechanism of the unique responsiveness of testicular germ cell tumor (TGCT) cells to cisplatin. PLoS One.

[88] Ehrlich Y, Baniel J (2007). Late relapse of testis cancer. Urol Clin North Am.

[89] Blagosklonny MV, Robey R, Sheikh MS, Fojo T (2002). Paclitaxel-induced FasL-independent apoptosis and slow (non-apoptotic) cell death. Cancer Biol Ther.

[90] Blagosklonny MV (2007). Mitotic arrest and cell fate: why and how mitotic inhibition of transcription drives mutually exclusive events. Cell Cycle.

[91] Bunz F, Hwang PM, Torrance C, Waldman T, Zhang Y, Dillehay L, Williams J, Lengauer C, Kinzler KW, Vogelstein B (1999). Disruption of p53 in human cancer cells alters the responses to therapeutic agents. J Clin Invest.

[92] Blagosklonny MV (2001). Paradox of Bcl-2 (and p53): why may apoptosis-regulating proteins be irrelevant to cell death?. Bioessays.

[93] Leontieva OV, Blagosklonny MV (2010). DNA damaging agents and p53 do not cause senescence in quiescent cells, while consecutive re-activation of mTOR is associated with conversion to senescence. Aging (Albany NY).

[94] Maki CG (2010). Decision-making by p53 and mTOR. Aging (Albany NY).

[95] Galluzzi L, Kepp O, Kroemer G (2010). TP53 and MTOR crosstalk to regulate cellular senescence. Aging (Albany NY).

[96] Wesierska-Gadek J (2010). mTOR and its link to the picture of Dorian Gray - re-activation of mTOR promotes aging. Aging (Albany NY).

[97] Blagosklonny MV (2007). Antagonistic drug combinations that select against drug resistance: from bacteria to cancer. Cancer Biol Ther.

